# Expression Pattern of Long Non-Coding RNAs in Renal Cell Carcinoma Revealed by Microarray

**DOI:** 10.1371/journal.pone.0099372

**Published:** 2014-06-06

**Authors:** Chao Qin, Zhijian Han, Jian Qian, Meiling Bao, Pu Li, Xiaobing Ju, Shaobo Zhang, Lei Zhang, Shuang Li, Qiang Cao, Qiang Lu, Jie Li, Pengfei Shao, Xiaoxin Meng, Wei Zhang, Changjun Yin

**Affiliations:** Department of Urology, the First Affiliated Hospital of Nanjing Medical University, Nanjing, China; University of Navarra, Spain

## Abstract

**Background:**

Recent large-scale transcriptome analyses have found large numbers of transcripts, including that of long non-coding RNAs (lncRNAs), which are aberrant in various diseases, especially cancers. However, it is not clear whether lncRNAs are involved specifically in renal cell carcinoma (RCC). We investigated the expression patterns of lncRNAs in five RCC tumor samples (T) relative to those of matched adjacent non-tumor tissues (N) via microarray.

**Methods:**

A microarray with 33,045 lncRNA probes and 30,215 mRNA probes was used to identify deregulated lncRNAs in five RCC patients. Furthermore, we confirmed the relative expression levels of AK096725 and ENST00000453068 in 70 paired samples by quantitative reverse transcription polymerase chain reaction (qRT-PCR).

**Results:**

The lncRNA microarray revealed 27,279 lncRNAs in RCC samples, of which 480 were significantly upregulated (*P*<0.05; T/N>1.5) and 417 were significantly downregulated (*P*<0.05; N/T>1.5) compared with the matched non-tumor samples. In addition, 19,995 mRNAs were detected, of which 458 were significantly upregulated (*P*<0.05; T/N>1.5) and 413 were significantly downregulated (*P*<0.05; N/T>1.5). The expression level changes of AK096725 (*P* = 0.043) and ENST00000453068 (*P*<0.001) in 70 paired samples were in accord with the microarray data.

**Conclusions:**

The study uncovered expression patterns of lncRNAs in 5 RCC patients, as well as a number of aberrant lncRNAs and mRNAs in tumor samples compared with the non-tumor tissues. The revelation of an association between AK096725 expression and RCC is especially noteworthy. These findings may help to find new biomarkers in RCC.

## Introduction

Over 70% of the human genome is found actively transcribed, but only 1–2% encodes proteins [1], [2]. Much of the remainder gives rise to long non-coding RNAs (lncRNAs) [3], [4]. The transcripts of lncRNAs contain more than 200 nucleotides and have little or no potential translation [4], [5]. Although once thought to have no function, the expression of these novel RNAs have been shown to be cell type-specific [6], [7], localized to sub-cellular compartments [8], [9], and associated with diverse human diseases including a number of cancers [10], [11].

Correlations between lncRNA expression and cancer has attracted worldwide research attention, as well the reported functions of lncRNAs in gene expression regulation [9], [12], splicing [13], epigenetic control [14], chromatin structure [15], [16], and nuclear transport [9]. Such correlations may be positive or negative. For example, the well-known lncRNA Hox antisense intergenic RNA (HOTAIR) is a powerful predictor of metastasis and poor prognosis, and is overexpressed in breast cancer, hepatocellular carcinoma (HCC) and colorectal cancer, to name a few [14], [17], [18]. Conversely, the lncRNA maternally expressed gene 3 (MEG3) is downregulated in HCC, and enforced expression of MEG3 in HCC cells significantly decreased both anchorage-dependent and anchorage-independent cell growth, and induced apoptosis [19]–[21].

Renal cell carcinoma (RCC) is one of the most common malignant cancers in China [22]. It was estimated that 37.7 men and 16.6 women per 100,000 Chinese were diagnosed with RCC in 2005 [22]. The disease is the third most common genitourinary cancer, and for the year 2008 in the United States 54,390 cases (predominantly male) and 13,010 deaths were expected [23]. Although in most patients RCC is primary, up to 40% will eventually develop metastases. Patients with metastatic RCC have a median survival of only 6–12 months and only 9% survive 5 years, largely because of strong resistance to chemotherapy and radiotherapy and the lack of effective therapeutics [24]. Thus methods for early detection and prognostic markers are required, as well as novel therapies. We were thus led to explore the potential role of lncRNAs in RCC.

To the best of our knowledge, there are only three works reporting microarray data on the expression of lncRNAs in RCC. In 2008, Brito et al. [25] first reported a subset of downregulated intronic noncoding RNAs in six patients with clear cell RCC (ccRCC). More recently the same group [26] performed another microarray experiment and uncovered a signature of differentially expressed intronic lncRNAs in 11 ccRCC patients. In 2012, Yu et al. [27] published the lncRNA expression signatures of six ccRCC patients determined through microarray.

In the present study, to identify aberrantly expressed lncRNAs in RCC we compared the microarray expression profiles of lncRNAs from cancer tissues of five RCC patients relative to those of matched healthy tissues. Two of the thousands of deregulated lncRNAs we identified were further evaluated in 70 pairs of matched tumor/non-tumor (T/N) tissues via quantitative reverse transcription polymerase chain reaction (qRT-PCR). Our results may help to find new biomarkers in RCC.

## Materials and Methods

### Ethics statement

The local Ethics Committees of the First Affiliated Hospital with Nanjing Medical University, Nanjing, China approved the study. All participants in the study provided written informed consent.

### Tissue samples

The tumor and adjacent non-tumor tissue specimens were obtained with informed consent from the RCC patients who underwent radical nephrectomy or partial nephrectomy at First Affiliated Hospital of Nanjing Medical University, China (Table 1, Table S1). All samples were taken during surgery, immediately frozen in liquid nitrogen, and stored at −80°C for further analysis. All the tumor and non-tumor tissue specimens were diagnosed histopathologically. Paired tumor and non-tumor tissues from five RCC patients were used for the microarray assay (Table 1) and from 70 RCC patients (including the 5 RCC patients used for microarray) for the qRT-PCR validation assay (Table S1).

**Table 1:** General information of the five male clear cell renal cell carcinoma patients for microarray.

Patient No.	Kidney	TMN stage	Tumor Size(cm^3^)	Surgical Method
1	Left	T1aN0M0	2×2×1.5	Laparoscopic partial nephrectomy
2	Right	T1bN0M0	5×4×4	Laparoscopic radical nephrectomy
3*	Left	T1aN0M0	3×2×2	Laparoscopic partial nephrectomy
4	Left	T1aN0M0	3.8×2×1.8	Laparoscopic partial nephrectomy
5	Left	T2aN0M0	7.5×7×5	Laparoscopic radical nephrectomy

*This patient had a history of right renal cell carcinoma and underwent radical nephrectomy of the right kidney.

### RNA extraction and lncRNA microarray analysis

Total RNA was extracted from tissues using TRIzol (Invitrogen), in accordance with the manufacturer's protocol, and purified by RNeasy Mini Kit (Qiagen). Total RNA from each sample was quantified and quality-assured by NanoDrop ND-1000. RNA integrity was assessed by standard denaturing agarose gel electrophoresis.

The sample preparation and microarray hybridization were performed based on the manufacturer's standard protocols with minor modifications. Briefly, mRNA was purified from total RNA after the removal of rRNA (mRNA-ONLY Eukaryotic mRNA Isolation Kit, Epicentre). Then, each sample was amplified and transcribed into fluorescent cRNA along the entire length of the transcripts without 3′ bias, utilizing a random priming method. The labeled cRNAs were hybridized onto the Human LncRNA Array v2.0 (8×60 K, Arraystar). After washing the slides, the arrays were scanned by the Agilent Scanner G2505B.

Agilent Feature Extraction software (version 11.0.1.1) was used to analyze the acquired array images. Quantile normalization and subsequent data processing were performed using the GeneSpring GX v12.0 software package (Agilent Technologies). The lncRNAs and mRNAs having at least 5 of 10 samples with flags in “Present” or “Marginal” were chosen for further data analysis. Kang Chen Bio-Tech, Shanghai P.R. China performed all the above work.

The microarray contained probes for 33,045 lncRNAs that were designed by Arraystar based on the most authoritative databases (RefSeq, UCSC Known genes, Ensembl and related literature); 30,215 coding transcripts were used for microarray assay in five RCC tissues and their matched non-tumor samples. Differentially expressed lncRNAs and mRNAs with statistical significance (*P*<0.05; T/N or N/T fold change [FC]>1.5) between the two groups were identified by comparing the normalized expression levels in tumor and non-tumor samples with a paired *t*-test. Then, hierarchical clustering was performed to make salient the diferential lncRNAs and mRNAs expression patterns. Furthermore, Gene Ontology (GO) and Pathway analyses were carried out, which may give us a glimpse at the microenvironment of the cancer and may help us explore the mechanism of the lncRNAs in RCC. The microarray data was deposited in the ArrayExpress database, and the accession number is E-MTAB-1830.

### qRT-PCR validation assay

Total RNA was reverse-transcribed into cDNA using a High Capacity RNA-to-cDNA Kit (Applied Biosystems) in accordance with the manufacturer's instructions. Real-time PCR was performed using a SYBR Select Master Mix (Applied Biosystems) protocol on an Applied Biosystems StepOnePlus Real-Time PCR system. The primers were: ENST00000453068 (5′-TCAATCCCTGAGAATCGCGG-3′, forward; 5′-CGATGTAGGCCGAGATCACC-3′, reverse); AK096725 (5′-TGCGCCTCCATACAGTTTGT-3′, forward; 5′-GAGGAGAGCAAGGGCAACTT-3′, reverse); β-actin, (5′-ACTGGAACGGTGAAGGTGAC-3′, forward; 5′-AGAGAAGTGGGGTGGCTTTT-3′, reverse). The reaction conditions were: 50°C for 2 min; 95°C for 2 min; and 40 cycles of 95°C for 15 s and 60°C for 60 s. All reactions were performed in triplicate and normalized by the internal control products of β-actin.

The median cycle threshold (Ct) of each triplicate was used to calculate relative lncRNA concentrations (ΔCt = Ct_lncRNA_−Ct_β-actin_). The fold change (FC) was determined using the comparative CT (2^−ΔΔCt^) method [28]. The lncRNA expression differences between the matched tumor and normal samples were analyzed using Student's paired *t*-test with SPSS software (version 13.0, SPSS). A probability value of *P*<0.05 was considered statistically significant.

## Results

### Differentially expressed lncRNAs

**Figure 1:**
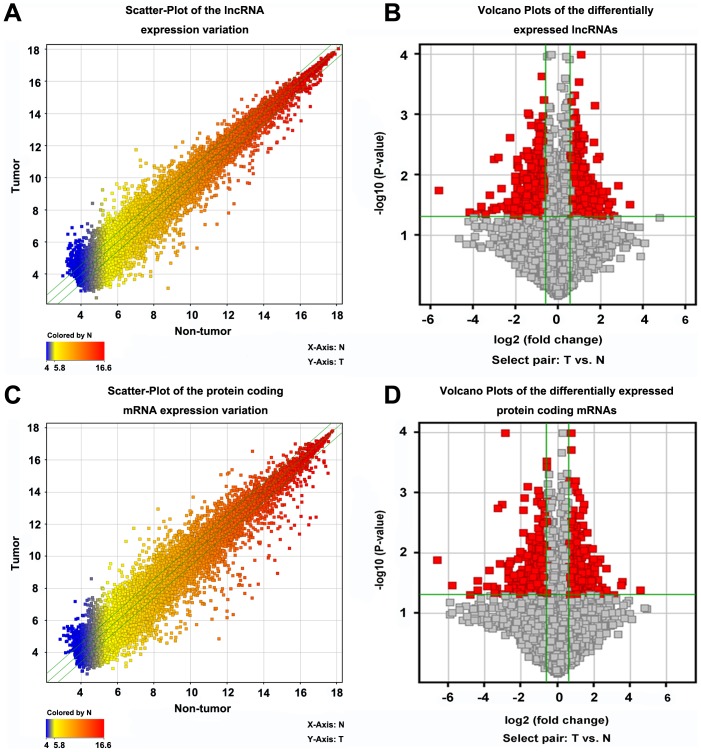
Overview of the microarray signatures. (A and C) Scatter-plots showing the variations in (A) the lncRNA and (C) protein-coding mRNA expressions between the tumor and non-tumor matched pairs of tissues. The values of the *X* and *Y* axes are the averaged normalized signal values of groups of samples (log_2_ scaled). The green lines are fold change lines (FC = 1.5). The color of the points indicates the intensities from low (blue) to high (red). The lncRNAs or mRNAs above the top green line and below the bottom green line indicated >1.5 FC between the two groups of samples. (B and D) Volcano plots of the differentially expressed (B) lncRNAs and (D) protein-coding mRNAs. The vertical lines correspond to 1.5 FC up and down and the horizontal line represents a *P*-value of 0.05. The red point in the plot represents the differentially expressed lncRNAs or mRNAs with statistical significance.

We obtained an overview of the aberrant lncRNAs by analyzing the microarray data (Figure 1A, 1B; Table 2). Significantly differentiated lncRNAs were defined as those with normalized expression levels consisting of fold changes greater than 1.5 (i.e., T/N>1.5 or N/T>1.5). From five paired samples we identified hundreds of significantly differentiated lncRNAs (Table S2). In total, there were 480 upregulated lncRNAs and 417 downregulated lncRNAs found in the 5 RCC patients. Specifically, the most upregulated lncRNAs were: uc001vjj.1, ENST00000414223, BC047917, uc003erl.1, and uc009wkz.1, of which uc001vjj.1 was the highest (log_2_FC = 3.367). The most highly downregulated were: ENST00000507950, uc001aka.2, NR_026860, NR_024256, and BC070168, of which ENST00000507950 showed the largest downregulation (log_2_FC = −5.611).

**Table 2:** A collection of the differentially expressed lncRNAs determined by microarray.*

Upregulated			Downregulated		
lncRNAs	Log_2_ fold change (T/N)	*P*-value	lncRNAs	Log_2_ fold change (T/N)	*P*-value
uc001vjj.1	3.367	0.031	ENST00000507950	−5.611	0.018
ENST00000414223	2.844	0.015	uc001aka.2	−4.186	0.041
BC047917	2.647	0.048	NR_026860	−3.823	0.044
uc003erl.1	2.526	0.027	NR_024256	−3.630	0.033
uc009wkz.1	2.466	0.047	BC070168	−3.401	0.044
DQ890550	2.381	0.031	NR_027130	−3.220	0.022
AY927487	2.335	0.039	CR613822	−3.104	0.039
uc004afh.2	2.256	0.019	AK022063	−3.026	0.006
NR_024373	2.116	0.024	AK094427	−3.015	0.035
ENST00000515243	2.113	0.024	ENST00000438623	−2.917	0.037
AK129874	2.097	0.048	ENST00000431789	−2.844	0.005
C20652	2.091	0.019	AK057998	−2.698	0.017
AL136790	1.963	0.027	uc004aww.1	−2.695	0.033
AK000957	1.927	0.047	NR_002942	−2.408	0.032
NR_024206	1.922	0.034	ENST00000431017	−2.406	0.035
ENST00000419196	1.911	0.005	ENST00000510795	−2.394	0.028
ENST00000449954	1.887	0.026	ENST00000453068	−2.365	0.047
ENST00000507775	1.873	0.037	BC150253	−2.285	0.030
uc003jgq.1	1.868	0.015	NR_024419	−2.270	0.002
ENST00000423390	1.854	0.035	uc001tfa.1	−2.209	0.034

*RCC tumor specimens (T) relative to adjacent non-tumor tissue samples (N), *P*<0.05.

Further analysis proceeded by classifying and stratifying the lncRNAs into subgroups, and deregulated subgroups of lncRNAs were revealed. Subgroups such as HOX lncRNAs and enhancer-like lncRNAs are thought to participate in numerous diseases such as cancers [29]–[32]. The HOX cluster profiling data showed all probes in the four HOX loci [15], targeting 407 discrete transcribed regions, lncRNAs, and coding transcripts (Table S3). But only 20 of 335 detected non-coding RNAs were significantly differentially expressed in RCC samples, and 12 of 207 coding transcripts were deregulated. In addition, we obtained profiling data of all probes for lncRNAs with enhancer-like function [33] (Table S4). We tested 1384 enhancer-like lncRNAs, and found that 33 of them were significantly upregulated, while 31 of them were significantly downregulated in RCC samples. Long intergenic non-coding RNAs (lincRNAs), such as the well-known HOTAIR, constitute a subgroup of the lncRNA family which is transcribed from intergenic regions. Based on John Rinn's papers [34], [35], 2256 lincRNAs were identified by microarray (Table S5, containing data of differentially expressed lincRNA and nearby coding gene pairs [distance <300 kb]). We found 57 lincRNAs were upregulated in RCC samples, and 47 were downregulated.

### Differentially expressed mRNA

In the five paired samples we detected 19, 995 mRNAs (Fig. 1C, 1D). Among them, 458 were significantly upregulated and 413 downregulated (*P*<0.05; FC>1.5) in the RCC samples (Table S6). The most significantly deregulated mRNAs were C10orf99 (upregulated) and AQP2 (downregulated).

### Validation of lncRNA expression

**Figure 2:**
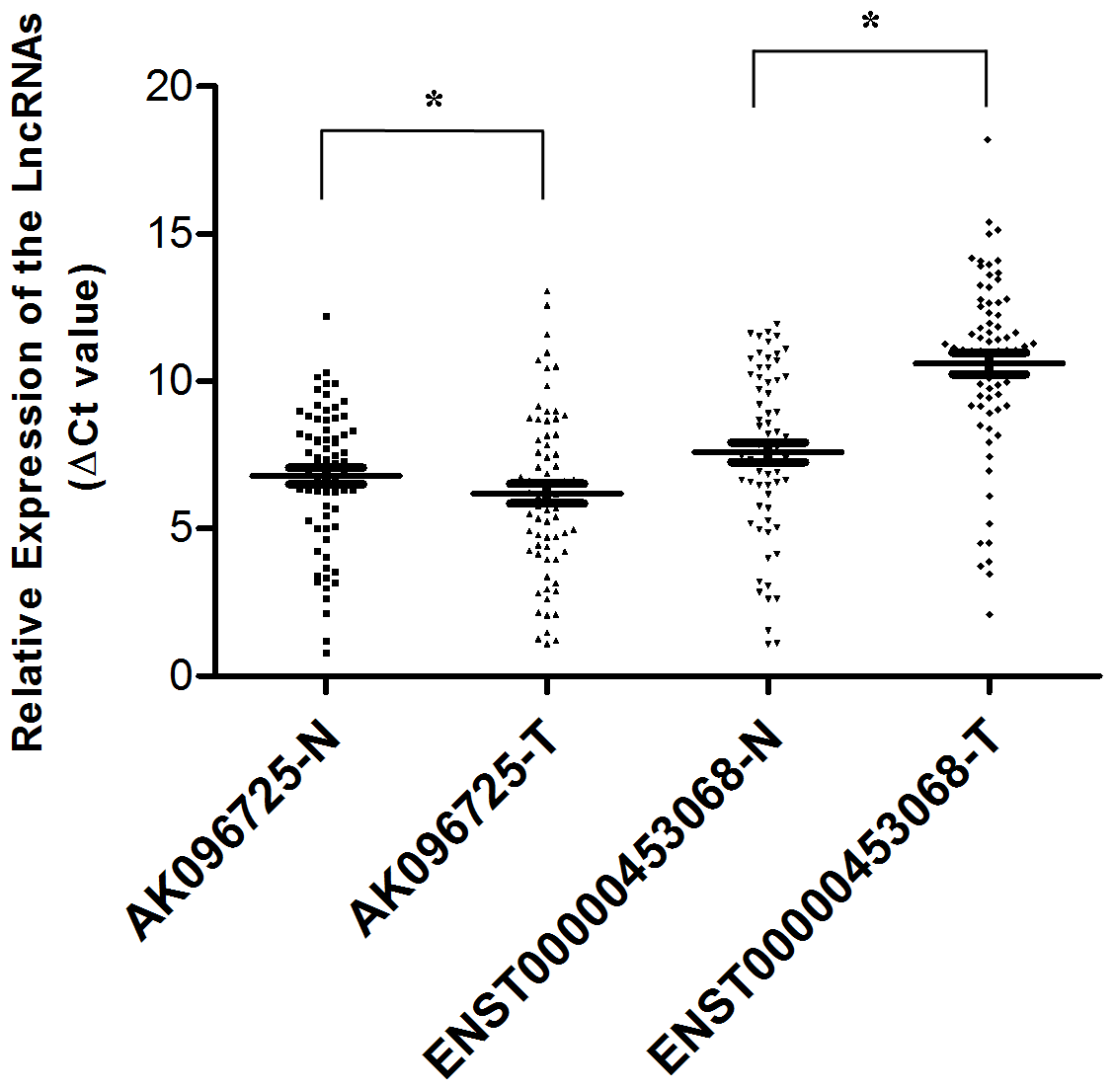
Relative expressions of AK096725 and ENST00000453068 in 70 paired RCC tumor specimens and adjacent normal tissue samples determined by qRT-PCR. The expression level of lncRNAs was normalized using β-actin as an internal control. The median in each triplicate was used to calculate the relative lncRNA concentration using the comparative ΔCt method. The lines are at mean with SEM. AK096725 was found significantly differentially upregulated in tumor specimens (*P* = 0.043), while ENST00000453068 was identified as significantly downregulated in tumor specimens (*P*<0.001).

To choose up- and down-regulated examples of lncRNAs to verify via qRT-PCR, we took note of differentially expressed lncRNAs and associated genes that have been implicated in the pathogenesis of cancer. We chose the lncRNAs AK096725 (upregulated) and ENST00000453068 (downregulated) to confirm their differential expression levels in 70 paired RCC tissues and adjacent non-tumor tissues. Levels of AK096725 were significantly greater (*P* = 0.043) in RCC tissues while those of ENST00000453068 was significantly lower (*P*<0.001; Figure 2) compared to the non-tumor tissues. These results are consistent with the microarray data.

**Figure 3:**
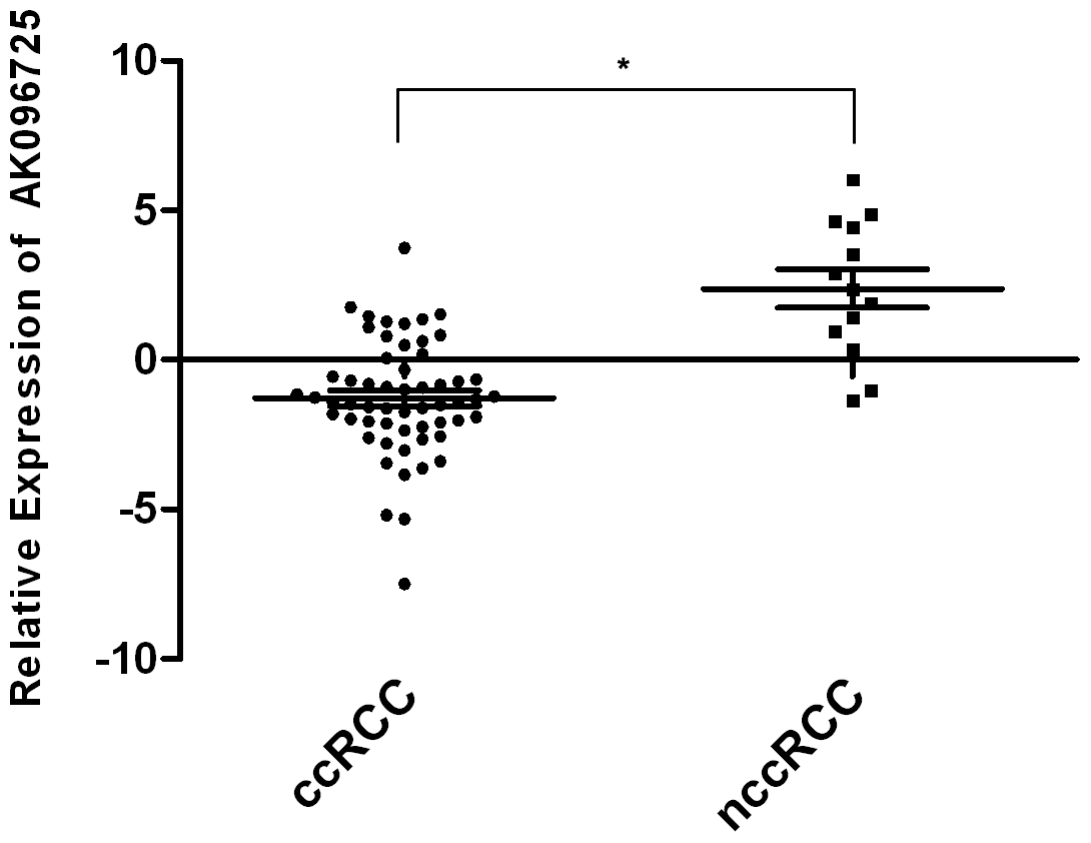
Analysis of AK096725 mRNA expression levels from the tumor and non-tumor tissues of ccRCC patients (57 cases) and nccRCC patients (13 cases), from qRT-PCR. β-actin was used as a control. Data is shown as fold change which is the normalized expression of tumor and non-tumor tissue from the same patient. The lines are at mean with SEM. The mRNA expression of AK096725 of ccRCC patients was significantly lower than that of the nccRCC patients (*P*<0.001).

Interestingly, we found that the differential expression of AK096725 was likely associated with the pathogenesis of RCC, because of the difference in fold changes from normal tissues between the ccRCC (57 cases; log_2_FC = −1.280) and the non-clear cell RCC (nccRCC; 13 cases; log_2_FC = 2.381; *P*<0.001; Figure 3). However, the sample size of the nccRCC being limited, a further study is required to verify whether the difference is pathologically specific. This dissimilarity was not noted in the lncRNA ENST00000453068 (*P* = 0.132, data not shown). We also analyzed the data by stratifying samples according to pathological stage. However, no differences in expression levels were found among tumor stages for either of the two lncRNAs (*P*>0.05, data not shown).

## Discussion

Over the past decades, the gene expression microarray has been recognized as a feasible and useful approach to profile the molecular signatures of RCC [24], [36]. Recently, a breathtaking number of lncRNAs have been discovered, and altered lncRNA expression patterns have been found associated with tumorigenesis and malignancy transformation in various cancers [37]. However, there are few lncRNAs reported in RCC. To uncover the expression pattern of lncRNAs in RCC, we investigated the lncRNA signatures of 5 RCC patients through an lncRNA microarray.

To identify differentially expressed lncRNAs which may function in RCC, we evaluated the lncRNA profiles of the RCC samples relative to adjacent non-tumor tissues. We then focused on the altered lncRNAs and validated the microarray data for two of them via qRT-PCR. There have been three previous reports of lncRNA profiles of ccRCC created through microarray [25]–[27]. The studies of Brito et al. [25] and Fachel et al. [26] focused on intronic lncRNAs and revealed 6 and 29 intronic lncRNAs respectively. Furthermore, Fachel et al. [26] identified 26 intronic lncRNAs significantly correlated with the five-year survival rates of RCC patients. However, intronic lncRNAs are only a fraction of the lncRNA family, and other lncRNA subgroups have yet to be identified. Furthermore, not all stages of RCC have been profiled—for example, the six patients whom Yu et al. [27] used for microarray were all at the American Joint Committee on Cancer clinical stage T1bN0M0. The samples used in the present study consisted of T1a, T1b, and T2a stages. One T1a sample had a history of radical nephrectomy because of RCC (right) one year previously that may indicate a more aggressive malignancy. Therefore our samples cover a wider range of stages and may better reflect disorders in RCC.

We also noted that few lncRNAs were deregulated in all or parts of previous studies [25]–[27]. This may be due, at least in part, to samples taken at different stages or differences in collecting samples. In addition, the patients in these three studies [22]–[24] were all pathologically diagnosed with ccRCC. This may help characterize ccRCC, but does not further understanding of RCC in general or delineate the differences in disparate pathological patterns. Therefore we analyzed 70 paired tissues that comprised different patterns of RCC, including clear cell, papillary, and chromophobe carcinomas, for two lncRNAs. Primarily, we divided the samples into a ccRCC group and an nccRCC group, and we found that fold changes in the levels of AK096725 relative to non-tumoral tissues were significantly different (*P*<0.001) between these two groups. Although other lncRNAs have been shown to be cell type-specific[6], [7], in light of the small sample size of the nccRCC group in the present study, a larger sample size and a study with more depth is required to make a determination of the specificity of AK096725.

AK096725 is a non-coding RNA from RNAdb [38] with a length of 1880 bp, and antisense to the coding genes *PCGF1* (polycomb group RING finger protein 1) and *LBX2* (ladybird homeobox 2). It seems reasonable that the lncRNA AK096725 might regulate *PCGF1* and *LBX2*, since we found that these protein-coding genes were significantly upregulated (*P* = 0.031 and *P* = 0.020 respectively) in the RCC samples (Table S6). *PCGF1* is reported to act as a transcriptional repressor of many genes, such as Hox genes [39], and may have a positive role in tumor cell growth by promoting cell cycle progression and enhancing cell proliferation [40], [41]. PCGF1 is a crucial component in the assembly of distinct polycomb repression complex 1 (PRC1)-related complexes, which may be involved in chromatin remodeling and modification of histones [42].

LBX2 is a transcription factor that is putatively expressed in the developing brain, eye, and urogenital system, including the gonadal tubercle, kidneys, and adrenal glands [43]. Recently, Beckedorff et al. [44] reported that the antisense lncRNA ANRASSF1 regulates the protein-coding gene expressed in the same genomic locus via recruitment of PRC2 and modification of the repressive H3K27me3 histone mark. Therefore, considering the close proximity of AK096725, *PCGF1*, and *LBX2* in the genome, we suggest that lncRNA AK096725 may take part in the regulation of PCGF1 and LBX2 and may have a role in the development of RCC.

ENST00000453068 is a processed transcript without a protein product, and has a length of 2957 bp, much more than 200 bp, and thus fits well with the definition for lncRNAs. The gene of ENST00000453068 is located at chromosome 7q21.2, and the protein-coding gene *CYP51A1* is close to it. *CYP51A1* encodes a member of the cytochrome P450 super family of enzymes, monoxygenases which catalyze many reactions involved in drug metabolism and synthesis of cholesterol, steroids, and other lipids [45]. The eventual involvement of lncRNA ENST00000453068 as a regulator of the neighboring gene *CYP51A1* in tumor cells in response to drugs could be the subject of future studies.

Although our study revealed the expression patterns and deregulation of many lncRNAs in RCC, their functions remain unknown. A boom in functional analyses has commenced in this emerging field, and there are recent reports of the characteristics and novel functions of these molecules [46], [47]. The diverse functions of lncRNAs include involvement in the integrity of the nuclear structure, regulation of gene expression, chromatin remodeling, transcription, and post-transcriptional processing. Yet our understanding of the functional role of lncRNAs is limited and further studies are needed to better understand the mechanisms through which these transcripts exert their function.

## Conclusions

This study revealed differential expression patterns of lncRNAs in 5 RCC patients, in which 480 upregulated and 417 downregulated lncRNAs were found in RCC tissues relative to matched normal tissues. In addition, we verified the differential expression levels of AK096725 and ENST00000453068 in 70 paired samples through qRT-PCR, which agreed with the microarray data. The possible association between AK096725 levels and RCC is especially noteworthy. These findings may help to discover new biomarkers in RCC.

## Supporting Information

Table S1 **Information of the 70 RCC patients for qRT-PCR validation assay.**
(DOC)

Table S2 **Differentially expressed lncRNAs.**
(XLS)

Table S3 **HOX cluster profiling.**
(XLS)

Table S4 **Enhancer lncRNA profiling.**
(XLS)

Table S5 **Rinn lincRNA profiling.**
(XLS)

Table S6 **Differentially expressed mRNAs.**
(XLS)
