# Supplementary material for: Expression Pattern of Long Non-Coding RNAs in Renal Cell Carcinoma Revealed by Microarray
Source: PLoS One. 2014 Jun 6;9(6):e99372. doi: 10.1371/journal.pone.0099372 (PMC4048223; doi:10.1371/journal.pone.0099372)
Supplement: Table S1 — Information of the 70 RCC patients for qRT-PCR validation assay. (DOC) [file pone.0099372.s001.doc]

**Table S1.** Characteristics of RCC Patients

| Characteristic |  | RCC |
| --- | --- | --- |
| Gender | Male | 43 |
|  | Female | 27 |
| Age, median (range) |  | 56 (25-80) y |
| T stage | T1 | 58 |
|  | T2 | 3 |
|  | T3 | 8 |
|  | T4 | 0 |
| Pathology | Clear cell renal cell carcinoma | 57 |
|  | Non-clear cell renal cell carcinoma | 13 |
|  | Renal papillary carcinoma | 5 |
|  | Chromophobe renal carcinoma | 4 |
|  | Others | 4 |
